# Cannabinol Regulates the Expression of Cell Cycle-Associated Genes in Motor Neuron-like NSC-34: A Transcriptomic Analysis

**DOI:** 10.3390/biomedicines12061340

**Published:** 2024-06-17

**Authors:** Alessandra Trainito, Agnese Gugliandolo, Luigi Chiricosta, Stefano Salamone, Federica Pollastro, Emanuela Mazzon, Maria Lui

**Affiliations:** 1 IRCCS Centro Neurolesi “Bonino-Pulejo”, Via Provinciale Palermo, Contrada Casazza, 98124 Messina, Italy; alessandra.trainito@irccsme.it (A.T.); agnese.gugliandolo@irccsme.it (A.G.); maria.lui@irccsme.it (M.L.); 2 Department of Pharmaceutical Sciences, University of Eastern Piedmont, Largo Donegani 2, 28100 Novara, Italy; salamone.ste@gmail.com (S.S.); federica.pollastro@uniupo.it (F.P.)

**Keywords:** cannabinol, cannabinoids, cell cycle, transcriptomic analysis, NSC-34

## Abstract

Cannabinoids are reported to have neuroprotective properties and play a role in neurogenesis and neuroplasticity in in vitro and in vivo models. Cannabinol (CBN) is a minor cannabinoid produced by the degradation of Δ^9^-tetrahydrocannabinol in *Cannabis sativa* L. and exhibits anti-oxidant, analgesic, anti-bacterial, and anti-inflammatory effects. In this study, we explored the biological effects of 20 µM CBN (6.20 µg/mL) on differentiated NSC-34 cells by MTT assay and next-generation sequencing analysis on the transcriptome. KEGG and Gene Ontology enrichment analyses have been performed to evaluate potential CBN-associated processes. Our results highlighted the absence of any cytotoxic effect of CBN. The comparative transcriptomic analysis pointed out the downregulation of *Cdkn2a*, *Cdkn2c* and *Cdkn2d* genes, which are known to suppress the cell cycle. *Ccne2*, *Cdk2*, *Cdk7*, *Anapc11*, *Anapc10*, *Cdc23*, *Cdc16*, *Anapc4*, *Cdc27*, *Stag1*, *Smc3*, *Smc1a*, *Nipbl*, *Pds5a*, *Pds5b*, and *Wapl* genes, renowned for their role as cell cycle progression activators, were instead upregulated. Our work suggests that CBN regulates the expression of many genes related to the cell cycle, which are required for axonal maturation, migration, and synaptic plasticity, while not affecting the expression of genes involved in cell death or tumorigenesis.

## 1. Introduction

*Cannabis sativa* L. is a plant of the Cannabaceae family whose use as a medicinal plant and as a textile fiber dates back to ancient times. Thanks to its abundance of phytochemical compounds, this plant is nowadays largely used in the pharmaceutical field [1]. *Cannabis sativa* L. contains more than 100 secondary metabolites, called phytocannabinoids, which are primarily produced in trichomes growing on female Cannabis inflorescences and in most aerial parts of the plant [2]. The concentration of these compounds is related to the variety, tissue type, age, growth, harvest time and storage condition of the plant [3,4,5]. Relative concentrations of cannabinoids found in different parts of the hemp plant have been extensively reported by Andre CM et al. [1].

Phytocannabinoids are known to affect the endocannabinoid system and to have a great biological effect on human health [6]. Some of them are indeed used for the treatment of several disorders, including insomnia, nausea, pain, fatigue, epilepsy and rheumatisms [7]. These compounds mainly act through the main receptors of the endocannabinoid system (ECS): cannabinoid receptor 1 (CB1) and cannabinoid receptor 2 (CB2). CB1s are mostly expressed in the central nervous system (CNS), while CB2s are mainly present in leukocytes [8]. These receptors belong to the family of G-protein receptors (GPCRs) and have many modulatory roles influencing several biological processes such as cell proliferation and differentiation [9]. Phytocannabinoids also activate the heat-sensitive vanilloid TRP channel family (TRPV), modulating pain and inflammation [10].

The major psychoactive compound isolated from Cannabis in the 1960s was identified as Δ^9^-tetrahydrocannabinol (Δ^9^-THC). Later on, other non-psychoactive phytocannabinoid compounds were isolated: cannabidiol (CBD), cannabigerol (CBG) and cannabichromene (CBC) [2]. These four molecules represent the main cannabinoids extracted from the Cannabis plant [11]. However, there are other minor cannabinoids with specific biological properties; among these, our study focused on cannabinol (CBN) and its biological role.

CBN was the first molecule identified in the *Cannabis sativa* L. in the 1930s. It is a Δ^9^-tetrahydrocannabinol degradation compound synthesized by oxidative reaction (caused by exposure to light, oxygen and heat) leading to the Δ^9^-double bond re-localization to form a fully aromatic molecule [12]. It has been demonstrated that CBN is an agonist of both CB receptors and acts as a regulator of TRPV2 channels [13]. Different studies reported CBN to act as an anti-oxidant [14], analgesic [15], anti-bacterial [16], and anti-inflammatory molecule [17]. Furthermore, it exerts a role in the regulation of cell cycle-associated genes through the PI3K/AKT signaling pathway [18].

Since minor cannabinoids are nowadays proposed as neuroprotective agents and are shown to enhance neurogenesis [19,20], we hypothesized that CBN promotes neural survival in motor neurons. Thus, we investigated the main genes and molecular processes affected by the treatment with this natural compound. In detail, the effect of CBN on motor neuron gene expression was assessed and further investigated using an in vitro model of this cell line.

We used NSC-34, a hybrid cell line obtained by the fusion of mouse neuroblastoma cells with motor neuron-enriched embryonic spinal cord cells. When differentiated in vitro, these cells show morphological and physiological characteristics associated with mature primary motor neurons [21] despite maintaining a proliferative trait [22]. Thus they represent a suitable model for studying the pathophysiology of motor neurons [23].

Data from the literature show that CBN can modulate the cell cycle [18]. The cell cycle consists of four phases: phase G1 (first gap), phase S (DNA synthesis), phase G2 (second gap) and phase M (mitosis). This process is governed by sequential expression of cell cycle proteins including cyclin-dependent kinases (Cdk), cyclins and cyclin-dependent kinase inhibitors (Cdki). This biological mechanism is essentially linked to cellular proliferation, cellular survival and cellular development. The fine regulation of proliferation and cell death is essential for the maintenance of cellular homeostasis [24]. During CNS development, after neuronal cells complete the proliferative process, they differentiate and withdraw from mitosis, entering a quiescent state (phase G0). Nevertheless, recent studies demonstrated the persistence of neurogenesis in the adult brain. The adult mammalian CNS has limited regenerative capacity, restricted in neurogenic niches where adult neural stem cells reside: the subventricular zone (SVZ) of the lateral ventricle and the subgranular zone (SGZ) of the hippocampal dentate gyrus [25,26]. Although differentiated neurons have lost their ability to proliferate, the proteins related to the cell cycle continue to be expressed. Recent studies have shown that these proteins could play a role in the mechanisms underlying brain plasticity and cellular survival [27].

Cyclin E, which is usually localized at the nuclear level in proliferating cells, was found in cytoplasm and enriched in dendrites and axons of cultured primary neurons. Odajima J. et al. observed the expression of Cyclin E in most anatomical regions in adult mouse brains and proposed an alternative function of this protein in postmitotic neurons: they demonstrated that the ablation of Cyclin E in differentiated cells led to a reduced number of synapses and dendritic spines [28].

Interestingly, other cell cycle-related proteins have shown roles not strictly associated with cell proliferation, including brain plasticity [29]. For example, recent studies have highlighted that the activity of Cdk7, a serine/threonine kinase, is critical for transcription and synaptic plasticity [30]. Moreover, while the anaphase promoting complex (APC) is highly expressed in postmitotic neurons in the adult brain [31], its role is still unclear, but this complex could be required for the regulation of axonal growth [32].

In this study, we administered CBN on differentiated NSC-34 cells, with the final aim of identifying a set of pathways or biological processes potentially disrupted by changes in gene expression caused by the compound. In particular, we focused on the neuron survival effect of CBN, at the concentration of 20 µM (6.20 µg/mL), that may be exerted through the modulation of cell cycle genes and their alternative functions.

## 2. Materials and Methods

### 2.1. Synthesis of CBN

*Cannabis sativa* L. was acquired from Canvasalus Srl (Monselice, Italy). A certified sample (Cs-CBD/03/2021) of the non-psychoactive material was stored in Novara laboratories. The plant belonged to chemotype III, the fiber hemp with CBD as major cannabinoid and a concentration of THC far below the 0.2% of yield and so not detected.

**Figure 1:**
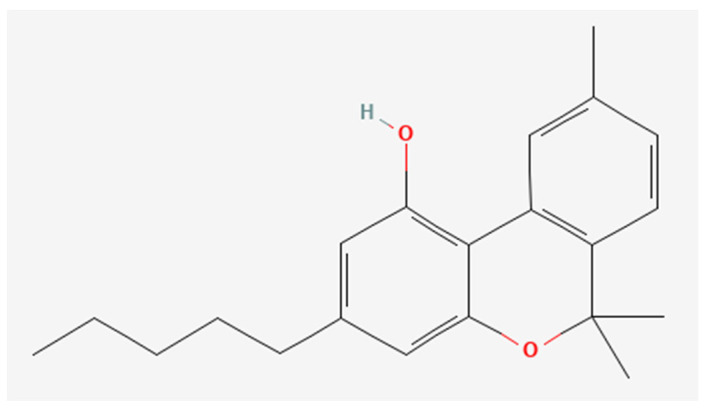
Chemical structure of CBN obtained by PubChem Compound Summary for CID 2543, Cannabinol. Retrieved 8 March 2024 [35], URL: https://pubchem.ncbi.nlm.nih.gov/compound/2543#section=2D-Structure&fullscreen=true.

CBD was obtained from the non-woody part of fiber hemp according to the procedure described by Pollastro et al. [33]. Specifically, iodine (162 mg, 0.64 mmol, 2 molar equiv.) was added to a solution of CBD (100 mg, 0.32 mmol) in toluene (20 mL). The solution was refluxed, following its course by TLC (petroleum ether/EtOAc, 90:10, Rf CBD = 0.55, Rf CBN = 0.45). After 60 min, the reaction was cooled to room temperature and sequentially washed with 5% Na_2_S_2_O_3_ and a saturated solution of NaCl in water. After drying, the organic phase was removed by evaporation, and the residue was purified by low-pressure chromatography on silica gel with petroleum ether 100% as eluent to afford 72 mg (72% yield) of CBN as a pale-yellow oil. The oil was further purified using HPLC JASCO Hichrom silica (petroleum ether/EtOAc 95:05, isocratic elution), obtaining 67 mg of CBN (99% purity). The pure CBN (Figure 1) was identified using ^1^H 400 MHz NMR spectra with Bruker 400 spectrometers (Bruker^®^, Billerica, MA, USA) as reported in Supplementary Material (Figure S1).

The metabolomic profile obtained by Choi et al. [34] was ultimately used as reference for the identification of CBN. Cannabinoids were identified with ^1^H 400 MHz NMR spectra with Bruker 400 spectrometers (Bruker^®^, Billerica, MA, USA), and chemical shifts were measured relative to the remaining solvent signal (CDCl_3_: *δ_H_* = 7.26). Silica gel 60 (70–230 mesh) for low-pressure chromatography was purchased from Macherey-Nagel (Düren, Germany). Purifications were monitored by staining with 5% H_2_SO_4_ in EtOH and subsequent heating by TLC. The chemical reagents and solvents employed were applied without any additional purification, unless specified. These reagents were purchased from Aldrich (Darmstadt, Germany). An HPLC JASCO Hichrom, 250 mm × 25 mm, silica UV–vis detector-2075 plus (Oklahoma, Japan) was used for the final purification of CBN.

### 2.2. NSC-34 Cell Culture and Treatment

The motor neuron cell line, NSC-34, provided by Cellutions Biosystem Inc., Cedarlane (Burlington, ON, Canada), was maintained in Dulbecco’s modified Eagle’s medium (DMEM) high glucose (#D5671, Sigma-Aldrich, St. Louis, MO, USA) supplemented with 1% L-glutamine (#G7513, Sigma-Aldrich, St. Louis, MO, USA), 1% penicillin/streptomycin (#P0781, Sigma-Aldrich, St. Louis, MO, USA) and 10% FBS (fetal bovine serum, not heat-inactivated) (#F7524, Sigma-Aldrich, St. Louis, MO, USA), at 37 °C in 5% CO_2_/95% air humidified atmosphere. Cells were subcultured every 2–3 days when cell density reached nearly 80–90% [36].

To induce the differentiation of NSC-34, the proliferation medium was replaced after 24 h by a differentiation medium containing 1:1 DMEM/F12 (Ham) (#D6421, Sigma-Aldrich, St. Louis, MO, USA), 1% L-glutamine, 1% penicillin/streptomycin, 1% FBS not heat-inactivated and 1 µM all-trans retinoic acid (atRA) (#R2625, Sigma-Aldrich, St. Louis, MO, USA). The cells were maintained for 5 days with the differentiation medium, which was renewed every 2 days [21].

After differentiation, NSC-34 cells were treated for 24 h with CBN at different concentrations (5, 10, 20, 50 µM). CBN was dissolved in dimethyl sulfoxide (DMSO) (#D8418, Sigma-Aldrich, St. Louis, MO, USA) in a 707.953 mM (Molecular Weight = 310.19 g/mol, equivalent concentration 219.6 × 10^3^ µg/mL) stock solution and diluted 1:2000 with phosphate-buffered saline 1 X (PBS) (#806552, Sigma-Aldrich, St. Louis, MO, USA) to an intermediate concentration of 353.97 µM before experimental use.

### 2.3. MTT Assay

CBN cytotoxicity was evaluated on differentiated NSC-34 cells through viability assay experiments. Specifically, an MTT quantitative colorimetric assay was carried out, since it allows measuring cellular metabolic activity as an indicator of cell viability, proliferation and cytotoxicity. The MTT colorimetric assay is based on the reduction of the soluble tetrazolium dye to its insoluble formazan, which can only be converted by viable cells. NSC-34 cells were plated at a density of 5 × 10^3^ cells per well in a 96-well microplate and then differentiated with 100 µL per well of differentiation medium containing 1 µM atRA for 5 days.

The differentiated motor neuron-like cells at 80–90% density were treated for 24 h with different CBN concentrations (5, 10, 20, 50 µM). Then, the medium was replaced with 100 µL of fresh medium supplemented with 0.5 mg/mL of MTT (Thiazolyl Blue Tetrazolium Bromide) (#M5655, Sigma-Aldrich, St. Louis, MO, USA) and incubated for 4 h at 37 °C in 5% CO_2_/95% air. Insoluble formazan crystals were dissolved in 100 µL of a 0.04 N HCl/isopropanol solution for 1 h [37]. The optical density was evaluated by spectrophotometry, and the absorbance at 570 nm was measured by a BioTek SynergyH1 microplate reader. Each experiment was performed with 8 repeats for each treatment group.

### 2.4. Library Preparation

NSC-34 cells were seeded in 6-well plates at a density of 1.5 × 10^5^ cells in 2 mL of medium per well, differentiated and treated with different concentrations of CBN (5, 10, 20, 50 µM). After 24 h, differentiated cells were harvested and pelleted for RNA extraction. Maxwell^®^ RSC simplyRNA Cells Kit (#AS1390, Promega, Madison, WI, USA) was used to extract the total RNA with a Maxwell^®^ RSC instrument. Library preparation was carried out with TruSeq^®^ RNA Exome protocol (Illumina, San Diego, CA, USA) [38], following the manufacturer’s instructions. A Tapestation 4150 instrument was used to validate the quality of the library with D1000 screentape (Agilent, Richardson, TX, USA). The Illumina instrument NextSeq 550Dx (Illumina, San Diego, CA, USA) was used to sequence the library using NextSeq 500/550 Mid Output Reagent Kit v2 (300 cycles), and the run was performed in paired-end mode.

### 2.5. Bioinformatics Analysis

Resulting raw paired-end reads from the NextSeq 550 Dx System were used to perform quality control assessment using FastQC (version 0.11.9) [39], which allows checking for the overall sequence quality, guanine-cytosine percentage distribution, sequence length distribution, overrepresented sequences and adapter content. Trimmomatic (version 0.40-rc1) [40] was used to perform the base clipping, adapter removal, trim for low-quality bases (at 3′ and 5′) and eventual filtering out of contaminants and low-quality regions.

STAR (Spliced Transcripts Alignment to a Reference) RNA-seq aligner (version 2.7.10a_alpha_220207) [41] allowed the alignment of the reads to the entire vM28 mouse reference genome from the GENCODE project (version M28, October 2021) [42] with high alignment sensitivity and precision. Aligned reads were quantified using HTSeq-count (version 0.13.5) [43], which preprocesses RNA-Seq data for differential expression analysis by counting the overlap of reads with the genes annotated in the reference genome vM28, comprehensive of both manual and evidence-based automated annotations.

Count data obtained with HTSeq-count were used as input for the DESeq2 (version 1.36.1) [44,45] R package (R version 4.2.0) to directly compare gene expression levels between samples treated with 20 μM CBN and control ones, and ultimately identify systematic changes between the two conditions. This R package was used to normalize the gene expression levels for each sample and to estimate the differential expression (upregulation or downregulation) calculated as fold changes (log_2_ ratio) according to the normalized gene expression levels in each sample, by the use of negative binomial. Differentially expressed genes (DEGs) were considered significant if their corresponding adjusted *p* values were ≤0.05, computed using the Benjamini–Hochberg method [46], meaning there was a significant difference in gene expression between the two samples.

Enrichment analysis of resulting DEGs was performed to investigate their functional roles and infer whether they are involved in common biological responses or perform related functions. Kyoto Encyclopedia of Genes and Genomes (KEGG, Release 109.0, 1 January 2024) [47] and Gene Ontology (GO release 17 January 2024) [48] were respectively used for pathway and GO term (Biological process, Molecular function and Cellular compartment) enrichment, setting a cutoff *p* value < 0.05 for significant enrichment. Specifically, GO and KEGG pathway enrichment analyses were performed using clusterProfiler Package (V.4.4.3) [49,50] of R (V.4.2.0) (R Core Team), part of the Bioconductor project for the analysis and interpretation of high-throughput data [51].

After Gene Ontology enrichment analysis, GOATOOLS Python library (V.1.4.9) [52] was used to obtain parents or ancestors for a subset of enriched GO terms and to plot GO hierarchies. Nested pie charts, nested bar plots, violin plots and chord diagrams were produced using matplotlib (V. 3.8.2) [53], seaborn (V. 0.13.2) [54] and d3blocks (V.1.1.5) [55] packages, with an in-house implemented script coded in Python (V 3.9.12) [56].

A comprehensive list of oncogenes and tumor suppressor genes was obtained by querying the Mouse Genome Database (MGD) [57], which collects data on mouse genes, their functions, phenotypes and mouse models of human disease. These data were used to investigate the role of the identified DEGs. Uniprot (release 2024-01) [58] was browsed to annotate the resulting cell cycle DEGs with their protein products and ultimately associate each gene with a specific cell cycle phase.

### 2.6. Immunocytochemistry

NSC-34 cells were plated on coverslips (10 mm, Epredia S.r.l., Milano, Italy) in 24-well plates at a density of 3.5 × 10^3^ in 500 µL of medium per well. The plates were pretreated with 0.1 mg/mL of poly-L-lysine hydrobromide (Sigma-Aldrich, Saint Louis, MO, USA) to improve cell adhesion. The cells were differentiated and treated with 20 µM CBN. After 24 h, cells were fixed with 4% paraformaldehyde (Santa Cruz Biotechnologies, Dallas, TX, USA) for 20 min at room temperature (RT), washed with 1X PBS and incubated at RT for 30 min with 3% H_2_O_2_ (Sigma-Aldrich, Saint Louis, MO, USA), suppressing the endogenous peroxidase activity. After 3 washes with PBS, the coverslips were blocked with blocking solution (1X PBS, 2.5% horse serum and 0.1% Triton X-100) for 20 min at RT according to the Vectastain^®^ Elite^®^ ABC-HRP kit (Vectastain, Glostrup, Denmark) protocol. Afterwards, they were incubated overnight at 4 °C with primary antibodies: CINtec p16 Histology (1.0 µg/mL; VENTANA^®^, Roche Diagnostics, Indianapolis, IN, USA) and anti-Cyclin D1 (0.1 µg/mL; VENTANA^®^, Roche Diagnostics, Indianapolis, IN, USA) for the evaluation of protein expression related to the cell cycle pathway. Then, the coverslips were washed 3 times with PBS and incubated with biotinylated secondary antibody (1:200; Vector Laboratories, Inc., Burlingame, CA, USA) and streptavidin AB Complex-HRP (ABC-kit from Dako, Glostrup, Denmark) for 30 min at RT.

The immunostaining was developed with the 3,3′-Diaminobenzedine (DAB) peroxidase substrate kit (Vector Laboratories, DBA Italia S.r.l., Milan, Italy; brown color; positive staining) and counterstained with nuclear fast red (Vector Laboratories, DBA Italia S.r.l.; pink background; negative staining). The images were captured using a light microscope (Axioscope 5 combined with axiocam 208 color camera; Zeiss, Oberkochen, Germany) equipped with an oil immersion objective 100× planabo. Densitometric analysis was performed using the software ImageJ (V.1.53).

### 2.7. Statistical Analysis

Data obtained were expressed as mean ± Standard Error of the Mean (SEM) and were analyzed by one-way ANOVA test and Bonferroni post hoc test for multiple comparisons for MTT assay or t test for immunocytochemistry analysis using GraphPad Prism software (version 9.5.1) (Boston, MA, USA). *p* values lower than 0.05 were considered significant.

## 3. Results

### 3.1. Evaluation of Cell Viability after CBN Treatment

**Figure 2:**
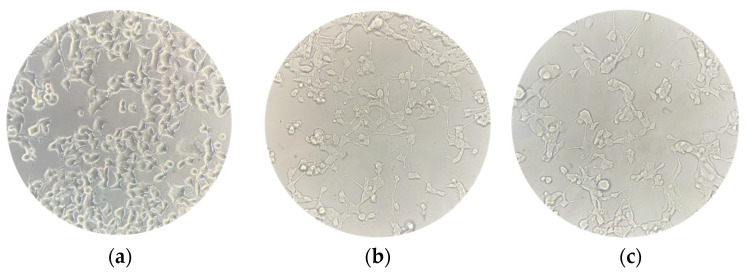
NSC-34 cell cultures were captured at three different timepoints to evaluate changes in their morphology. (**a**) Undifferentiated NSC-34 cells; (**b**) differentiated NSC-34 cells; (**c**) differentiated NSC-34 cells treated for 24 h with 20 μM CBN. Scale bar = 20 μm, 40× magnification.

NSC-34 cells were differentiated for 5 days into motor neuron-like cells (differentiated NSC-34 cells are shown in Figure 2). Afterwards, the differentiated cells were treated for 24 h with different concentrations of CBN in the range of 5 µM to 50 µM.

**Figure 3:**
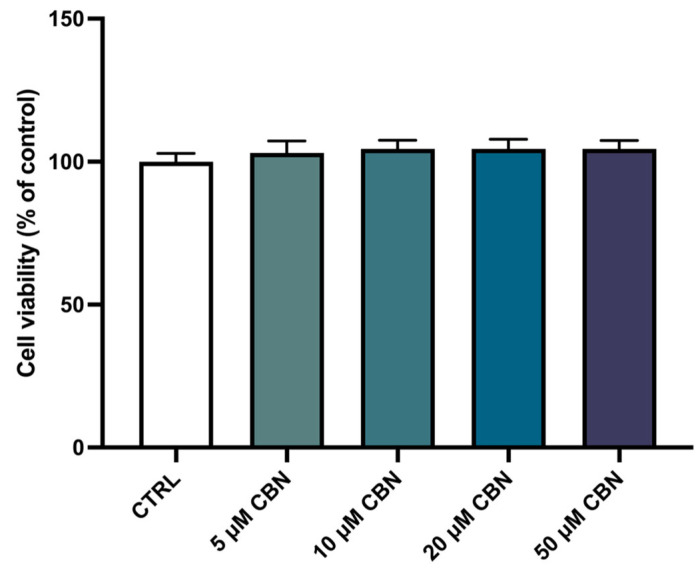
Cell viability after CBN treatment for 24 h at different concentrations (5, 10, 20, 50 µM) on differentiated cells compared to untreated differentiated NSC-34 cells (CTRL). The data represent the eight replicates’ means ± SEM. One-way ANOVA and Bonferroni post hoc test showed no significant differences (with *p* value lower than 0.05) between the treated samples and the control ones.

The spectrophotometric absorbance resulting from the MTT viability assay did not highlight any significant difference between the untreated differentiated NSC-34 cells (CTRL) and the differentiated NSC-34 cells treated with different concentrations of CBN (5, 10, 20 and 50 µM). As reported in Figure 3, CBN did not decrease cell viability at tested concentrations. According to our results, the compound is not cytotoxic, even at the highest tested concentration. Figure 2c reports the morphology of differentiated NSC-34 cells treated with 20 µM of CBN.

### 3.2. Transcriptomic Analysis

The comparative expression analysis between the differentiated NSC-34 cells treated with CBN for 24 h at different concentrations (5, 10, 20, 50 µM) and untreated differentiated NSC-34 cells (CTRL), followed by pathway enrichment analysis, revealed the enrichment of the cell cycle pathway for all the tested concentrations when compared to CTRL.

Among the DEGs produced from each comparison (5 µM CBN vs. CTRL, 10 µM CBN vs. CTRL, 20 µM CBN vs. CTRL and 50 µM CBN vs. CTRL), 31 were shared across all the tested CBN concentrations. For the sake of completeness, we have provided the Supplementary Figure S2 showing the heatmap for the genes that were differentially expressed at each CBN concentration. These 31 DEGs showed a coherent pattern, meaning each gene was either up- or downregulated in all comparisons.

**Figure 4:**
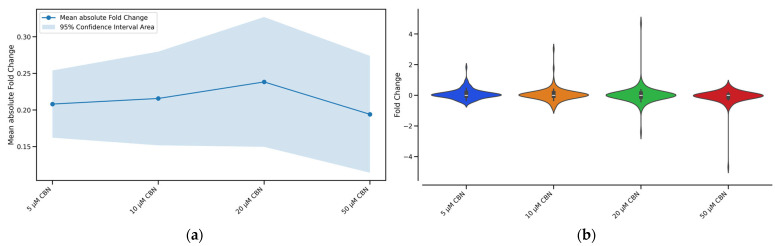
Graphical representation of the effect of the different CBN concentrations (5 µM, 10 µM, 20 µM, and 50 µM) on the expression of cell cycle genes after 24 h of incubation: (**a**) Line plot with confidence intervals representing the overall effect of the different CBN concentrations on cell cycle pathway genes. The blue solid line represents the fold change absolute values’ mean for each concentration, while the shaded light blue area around the mean line indicates the 95% confidence intervals. Treatment with 20 µM CBN had the highest mean fold change of absolute values. (**b**) Violin plot shows the shape of the distribution of the cell cycle genes’ fold changes for each tested CBN concentration. Treatment with 20 µM CBN was characterized by DEGs with more extreme fold changes than seen with other concentrations.

To determine the CBN concentration producing the greatest perturbation of the cell cycle pathway, we evaluated the number of enriched Biological Processes (BP) related to cell cycle together with fold changes’ absolute mean and distribution. The concentrations of 5, 10, 20 and 50 µM CBN were respectively enriched by 42, 43, 45 and 43 BP related to cell cycle. The overall effect caused by the compound at different concentrations (5, 10, 20, 50 µM) on every gene belonging to the cell cycle pathway is reported in Figure 4. For each tested CBN concentration, we report in Figure 4a the fold change absolute values’ mean and in Figure 4b the distribution of the cell cycle genes’ fold changes. Differentiated NSC-34 cells treated with the cannabinoid (20 µM CBN) had the highest mean fold change (reported as absolute values) and overall, the utmost values of fold change. Therefore, this concentration was selected to further investigate the effect of the compound on cell cycle-related genes.

The differential expression analysis carried out on 20 µM CBN-treated cells vs. CTRL produced 5569 differentially expressed genes (DEGs), 2836 upregulated and 2733 downregulated.

The enrichment pathway analysis carried out with clusterProfiler produced 102 enriched pathways in 20 µM CBN-treated cells compared to CTRL. Among the most enriched KEGG pathways, we focused on the cell cycle (mmu04110), since it was enriched by 78 DEGs (40 upregulated and 38 downregulated), with an adjusted *p* value of 1.027 × 10^−9^. Of these, 74 encoded for a protein involved in a specific phase of the cell cycle.

**Figure 5:**
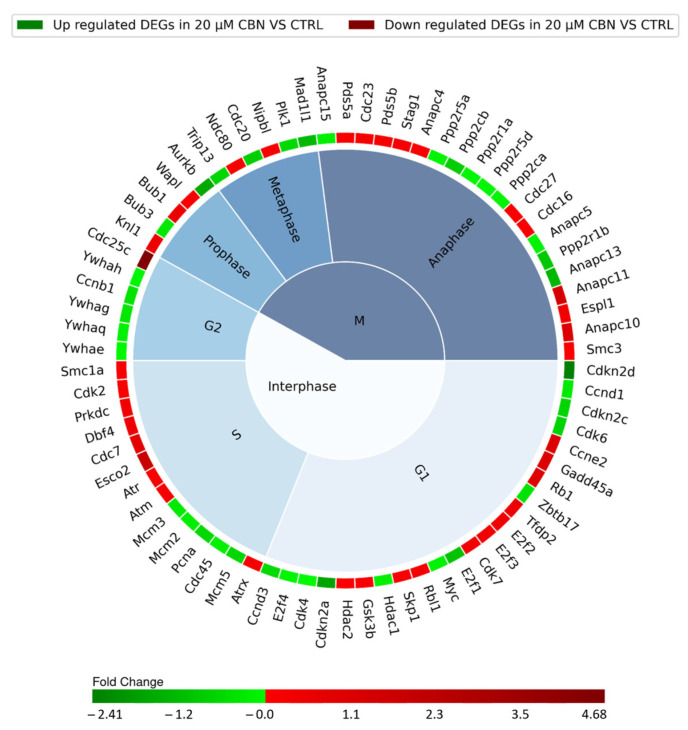
Nested pie chart represents the DEGs in cell cycle KEGG pathway (mmu04110), resulting from the comparison of 20 µM CBN-treated cells vs. CTRL. The chart is divided into the different phases of the cell cycle, and the DEGs were associated with a specific phase using Uniprot. The red boxes indicate the upregulated genes, and the green ones the downregulated genes. A color map of the fold changes for each DEG indicates the magnitude of differential expression for each gene (schematically reported in Table S2).

The cell cycle comprises multiple phases classified into Interphase and phase M, consisting of three and four subphases, respectively. The G1, S, and G2 subphases form the Interphase while prophase, metaphase, anaphase and telophase make up phase M. Cell cycle progression along the different phases is regulated by the sequential expression, inhibition and activation of many different proteins encoded by a specific set of genes. Each resulting DEG of the cell cycle pathway was therefore associated with a specific cell cycle phase (Figure 5), according to Uniprot database protein function annotation.

The interphase is characterized by 43 DEGs: specifically, 11 upregulated and 12 downregulated genes involved in G1 phase, 9 upregulated and 5 downregulated genes encoding for proteins taking action during phase S, and 1 upregulated gene and 5 downregulated genes involved in G2 phase. Thirty-one different genes encoding for proteins regulating the mitotic phase (phase M) were differentially expressed: 16 were upregulated and 15 were downregulated (Figure 5). The remaining four DEGs of the 78 related to the cell cycle pathway were not associated with any specific phase of the cell cycle. They actually encode for upstream regulators of the cell cycle: *Tgfb2* and *Smad3*, which were upregulated, and *Smad4* and *Crebbp*, which were downregulated.

The comparative expression analysis revealed a downregulation of cyclins (*Ccn*) and cyclin-depended kinase (*CdK*) involved in the early G1 phase (as reported in Figure 4 for *CCnd1*, *Ccnd3*, *Cdk4*, *Cdk6*, and *Cdk7*). Cyclin-dependent kinase inhibitor genes *Cdkn2a*, *Cdkn2c* and *Cdkn2d* were downregulated. *Rb1* gene, which encodes for the retinoblastoma-associated protein, was upregulated. Transcription factors *E2fs* were both up- and downregulated. G1/S progression seems to have been promoted, since it is regulated by the association of *Ccne2* and *Cdk2*, which were upregulated.

The genes associated with DNA replication showed a negative regulation (*Cdc45*, *Mcm2*, *Mcm3* and *Pcna*), except for *Dbf4* and *Cdc7*. Moreover, genes involved in DNA damage repair (*Atm*, *Atr*, *Atrx*, *Esco2*, *Gadd45* and *Prkdc*) showed increased expression, suggesting their active role during DNA synthesis. Our expression analysis highlighted an upregulation of *Cdc25c* with a downregulation of *Ccnb1*. The proteins encoded by these two genes are involved in G2/M transition regulation through the dephosphorylation of Cyclin B/Cdk1 complex by phosphatase Cdc25. The genes encoding for proteins belonging to the 14-3-3 family, *Ywhae*, *Ywhag*, *Ywhah* and *Ywhaq*, which inhibit the interaction between Cdc25 and Cyclin B/Cdk1, were downregulated [59].

We found several dysregulated genes linked to chromatid segregation during phase M: *Bub1*, *Espl1*, *Knl1*, *Ndc80*, *Nipbl*, *Pds5a*, *Pds5b*, *Smc1a*, *Stag1* and *Wapl* were upregulated; and *Aurkb*, *Bub3* and *Cdc20* were downregulated. Serine/Threonine phosphatase genes (*Ppp2r5a*, *Ppp2cb*, *Ppp2r1a*, *Ppp2r5d*, *Ppp2ca* and *Ppp2r1b*) were all downregulated. The genes coding for the members of Anaphase-promoting complex (APC/C complex) (*Anapc11*, *Anapc10*, *Cdc23*, *Cdc16*, *Anapc4*, *Cdc27* and *Smc3*) were instead upregulated, except for *Anapc5*, *Anapc15* and *Anapc13*, which were downregulated. Mitotic arrest deficient 1 like 1 gene (*Mad1l1*) was downregulated.

### 3.3. Enriched Gene Ontologies (GO)

**Figure 6:**
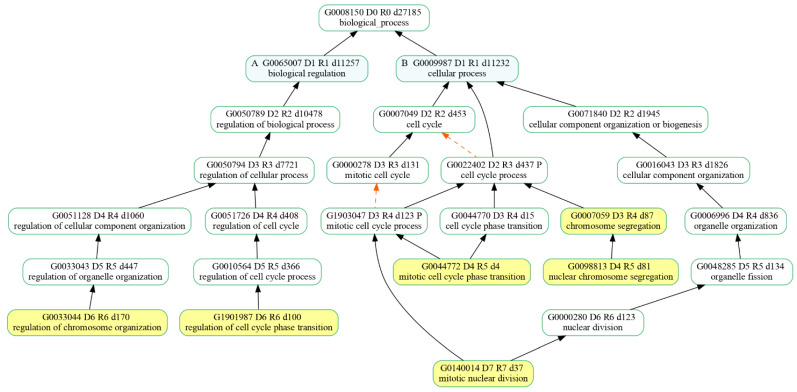
Hierarchy of the selected biological processes (highlighted in yellow) that were enriched in our comparative expression analysis of 20 µM CBN-treated samples vs. control ones. “is_a” relationship is specified by solid black arrows while “part_of” relationship is indicated by red dash arrows.

Since we were interested in the cell cycle pathway, we looked for enriched biological processes (BPs) dealing with cell proliferation among the Enriched Gene Ontologies (GO) from the clusterProfiler analysis of the identified DEGs. Among the 30 most significant biological processes in terms of *p* value, six of them were associated with biological processes taking part in the cell cycle main pathway (Figure 6). Specifically, the resulting BPs were as follows: regulation of chromosome organization (GO:0033044), regulation of cell cycle phase transition (G:1901987), mitotic nuclear division (GO:140014), mitotic cell cycle phase transition (GO:0044772), nuclear chromosome segregation (GO:0098813) and chromosome segregation (GO:0007059).

**Figure 7:**
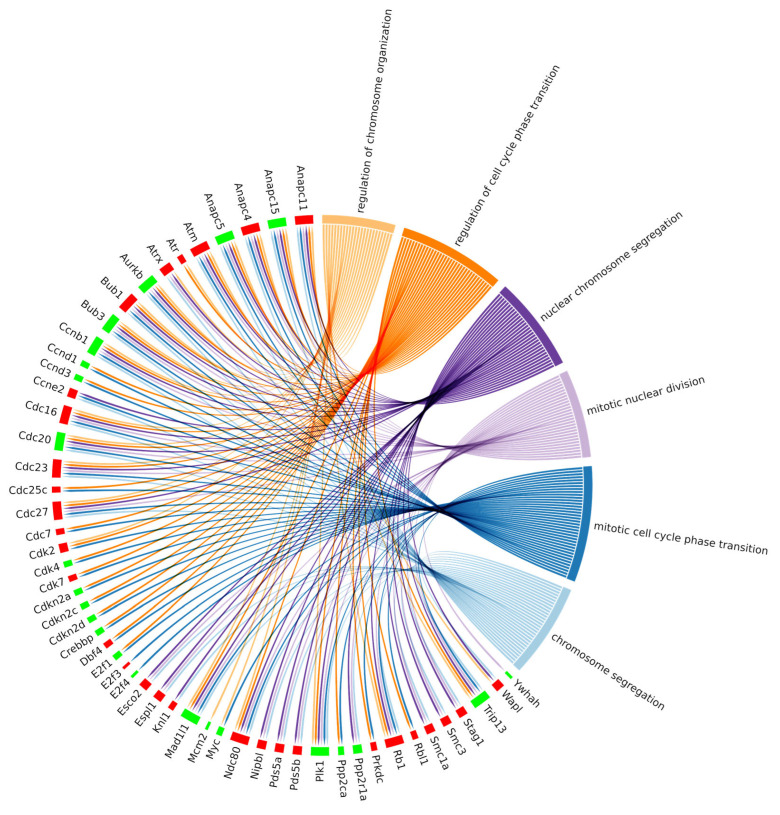
Chord plot representing the linkages of the differentially expressed genes of the KEGG cell cycle pathway and biological processes obtained among the top 30 biological process GOs with the lowest adjusted *p* value related to the cell cycle. The red boxes indicate the upregulated genes, and the green ones the downregulated genes.

Additionally, we investigated whether the DEGs belonging to the cell cycle pathway were also present in the enriched ontologies. This relationship has been reported in the chord plot (Figure 7), where cell cycle DEGs also belonging to at least one BP enriched ontology are summarized. Interestingly, 53 out of 78 DEGs of the cell cycle pathway were linked to significant biological processes, as reported in Table 1, which summarizes the DEG count for each enriched biological process under investigation. Specifically, we reported six different gene ontology terms comprising a total of 53 differentially expressed genes, divided into 30 upregulated and 23 downregulated.

**Figure 8:**
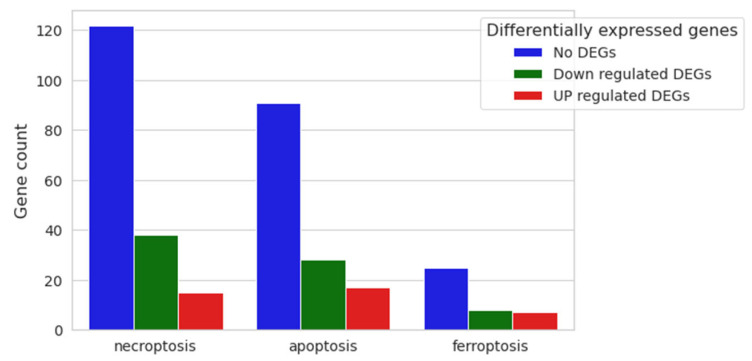
Nested bar plot of gene count for necroptosis, apoptosis and ferroptosis pathways, with differential expression resulting from 20 μM CBN vs. CTRL comparative analysis. For each cell death process, the number of downregulated, upregulated and not differentially expressed genes in 20 μM CBN are shown.

Since our expression analysis results involved cell cycle-related DEGs, we decided to look for possible enriched cell death processes, although our vitality assay did not reveal any mortality at the tested CBN dose. Therefore, the three main cell death pathways were investigated: necroptosis (mmu04217), apoptosis (mmu04210) and ferroptosis (mmu04216) (Figure 8), highlighting no activation of any of them.

Necroptosis is characterized by 175 genes, of which 122 were not differentially expressed in our comparative expression analysis (No DEGs); 38 were downregulated, and only 15 were upregulated genes (Supplementary Table S4). Apoptosis is characterized by 181 genes: 136 of them were not differentially expressed between the two conditions, 28 were downregulated, and only 17 were upregulated (Supplementary Table S4). Finally, the ferroptosis pathway is composed of 40 genes: 25 were not differentially expressed (No DEGs), 8 were downregulated and only 7 were upregulated (Supplementary Table S4).

**Figure 9:**
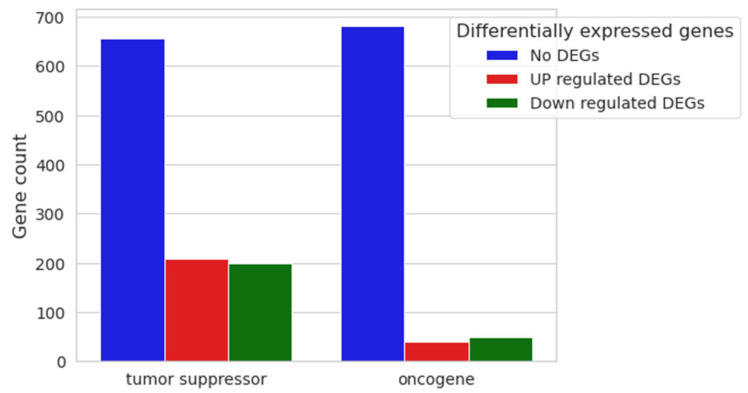
Nested bar plot of 20 μM CBN vs. CTRL differential expression analysis. Gene counts of both tumor suppressors and oncogenes are represented as nested bar plots. Relative non-differentially expressed genes and upregulated and downregulated gene counts in 20 μM CBN are shown for each category.

With the final aim of inferring whether or not the cell cycle dysregulation could eventually trigger an oncogenic process, we evaluated the differential expression of the main oncogenes and tumor suppressors deposited in publicly available databases. Genes associated with neoplastic formation, both oncogenes and tumor suppressors, were inspected using Mouse Genome Informatics (MGS), resulting in 773 and 1064 genes, respectively, with dysregulation as shown in Figure 9.

Among the resulting 1064 genes classified as tumor suppressors, 656 were found to have no differential expression in our comparative analysis, 208 were upregulated, and 200 were downregulated. Among the 773 annotated oncogenes derived from MGS, 683 were not differentially expressed in the comparison, 41 showed upregulation, and 49 showed downregulation (Supplementary Table S5).

### 3.4. Immunocytochemistry

**Figure 10:**
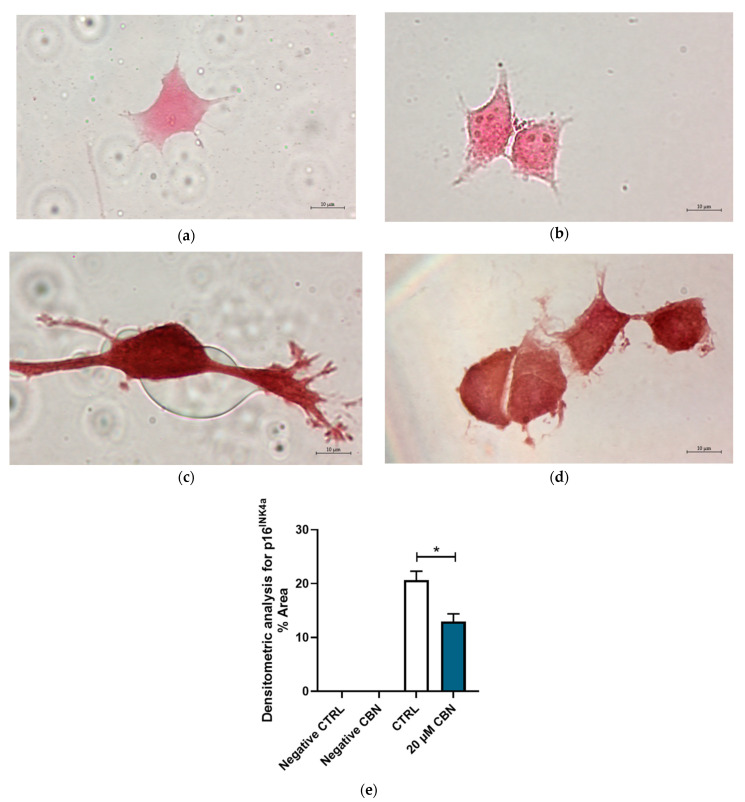
Immunocytochemistry analysis of differentiated NSC-34 cells. Untreated differentiated NSC-34 cells without primary antibody (Negative CTRL) (**a**), 20 µM CBN-treated differentiated NSC-34 cells without primary antibody (20 µM CBN Negative CTRL) (**b**), untreated differentiated NSC-34 cells immunoprofiled for p16^INK4a^ (**c**), and 20 µM CBN-treated differentiated NSC-34 cells immunoprofiled for p16^INK4a^ (**d**). Objective: 100×. Densitometric analysis for p16^INK4a^ (**e**). The data represent the percentage of staining resulting from the immunohistochemistry analysis. We report the mean ± SEM of the samples. Statistical significance was evaluated by *t* test. * indicates a significant difference (*p* < 0.05) between p16 ^INK4a^ expression level in 20 µM CBN compared to CTRL.

The protein expression of p16^INK4a^ and Cyclin D1 was evaluated by immunocytochemistry. Figure 10 shows the differential expression of cell cycle inhibitor p16^INK4a^ between 20 μM CBN and CTRL. p16^INK4a^ expression levels resulting from the transcriptomic analysis were confirmed by the densitometric analysis of the immunocytochemistry assay. Greater expression of this protein was noticeable in untreated differentiated NSC-34 cells compared to 20 µM CBN-treated differentiated NSC-34 cells (Figure 10c–e).

**Figure 11:**
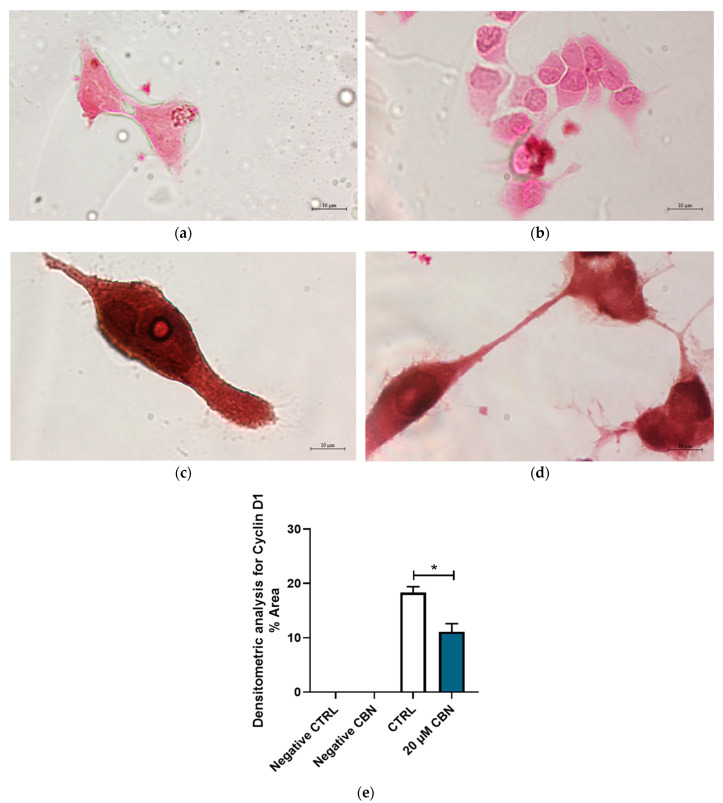
Immunocytochemistry analysis of differentiated NSC-34 cells. Untreated differentiated NSC-34 cells without primary antibody (Negative CTRL) (**a**), 20 µM CBN-treated differentiated NSC-34 cells without primary antibody (20 µM CBN Negative CTRL) (**b**), untreated differentiated NSC-34 cells immunoprofiled for Cyclin D1 (**c**), and 20 µM CBN-treated differentiated NSC-34 cells immunoprofiled for Cyclin D1 (**d**). Objective: 100×. Densitometric analysis for Cyclin D1 (**e**). The data represent the percentage of staining resulting from the immunohistochemistry analysis. We report the mean ± SEM of the samples. Statistical significance was evaluated by *t* test. * indicates a significant difference (*p* < 0.05) between Cyclin D1 expression level in 20 µM CBN compared to CTRL.

In addition, Figure 11 highlights the differential expression of Cyclin D1 between 20 μM CBN and CTRL. Cyclin D1 was expressed in both conditions; however, its expression levels in 20 µM CBN-treated differentiated NSC-34 cells were lower than in the untreated ones (Figure 11c,d). This observation was confirmed by densitometric analysis, which evidenced a significant difference in Cyclin D1 concentrations between 20 µM CBN and CTRL samples (Figure 11e).

## 4. Discussion

*Cannabis sativa* L. has been used as a medicinal plant for a thousand years, and its biological role in human health is still a subject of study [60]. Current research on therapeutic cannabis is mainly focused on the major phytocannabinoids, such as Δ^9^-tetrahydrocannabinol (Δ^9^-THC) and Cannabidiol (CBD), while the effect of minor phytocannabinoids on health is still mostly unknown [61].

Since minor cannabinoids have been recently proposed as neuroprotective compounds and are also known to induce neurogenesis in adult brain [19,20], we decided to focus our study on an investigation of the biological effect of Cannabinol (CBN). It has been demonstrated that CBN has a neuroprotective role in an in vitro model of Alzheimer’s disease [62]. Moreover, a transcriptional analysis demonstrated CBN to attenuate alterations in mitochondrial dynamics in an in vitro model of Parkinson’s disease (PD) [63]. Although CBN appears to have neuroprotective effects, to our knowledge, there is no evidence yet on the role of CBN as treatment for neurodegenerative diseases.

Differentiated NSC-34 cells express several motor neuron-like properties, which has led to the wide use of these cells as a suitable in vitro model for studying the pathophysiology of motor neurons characterized by progressive motor neuron cell death [64]. Unlike other cell types, neurons are very specialized cells, terminally differentiated as they exit the cell cycle and remaining permanently in the G0 phase. Despite that, many cell cycle-related proteins have been found to be constitutively expressed in postmitotic neurons. Several studies reported the adult brain to actively express the genes coding for those proteins. Their expression in physiological conditions has been related to noncanonical roles fulfilling differentiative functions such as neuronal maturation, neuronal migration and synaptic plasticity [27,28]. On the other hand, in pathological conditions, neurons upregulate the expression of cell cycle-related proteins and try to re-enter the cell cycle. This may result in the premature end of the cell cycle in the G1/S phase, inducing cell death by the activation of the apoptosis pathway [65,66].

After treatment with CBN, the viability of differentiated NSC-34 cells was assessed, and the substance showed no toxicity at tested concentrations (5, 10, 20, 50 µM) (Figure 3).

Since no significant decrease in cell viability was detected, we decided to analyze the transcriptomic profiles of NSC-34 cells treated with different concentrations of CBN to eventually understand the response of motor neuron-like cells to the phytocompound.

The comparative expression analysis on differentiated NSC-34 cells treated with CBN (5, 10, 20, 50 µM) vs. CTRL prompted our choice to further investigate the effect of 20 μM CBN since it was the concentration that had the largest effect on the cell cycle pathway genes. Specifically, as reported in Figure 4, the concentration of 20 μM CBN was characterized by the highest mean fold change (reported as absolute values). This suggested the cell cycle pathway was more impacted by the treatment with 20 μM CBN compared to the other tested concentrations.

The transcriptomic analysis of the comparison of 20 μM CBN vs. CTRL produced 102 enriched pathways. Among the most enriched KEGG pathways, we focused on cell cycle (mmu04110), since it was enriched by 78 DEGs (40 upregulated and 38 downregulated). Afterwards, we looked for enriched biological processes (BP) related to the cell cycle pathway, and we selected the most significant ones. We identified the top 30 BPs in terms of *p* value, and six of them were associated with cell cycle functions: specifically, regulation of chromosome organization (GO:0033044), regulation of cell cycle phase transition (GO:1901987), mitotic nuclear division (GO:140014), mitotic cell cycle phase transition (GO:0044772), nuclear chromosome segregation (GO:0098813) and chromosome segregation (GO:0007059).

These processes are complex and are characterized by several regulatory factors controlling their function. The significant DEGs we found associated with these ontologies promote their activation; however, a definite net effect on the entire GO is difficult to evaluate. Interestingly, these DEGs have been associated with synaptic plasticity and cell survival in postmitotic neurons [27].

Therefore, we independently examined the genes linked to the cell cycle pathway for potential alternative roles. Additionally, although our vitality assay did not reveal any mortality, we further investigated any possible cell death process regulated by the resulting DEGs. The cell cycle is a progression of a specific sequence of events directed by numerous regulatory proteins through the stages G1, S, G2 and M. During the cell cycle, cells execute two basic functions during cell division: replication of the genetic material (phase S) and partitioning of all the cellular components between two identical cells (phase M). The other two phases of the cell cycle, G1 and G2, represent the ones preceding the S and M phases, respectively. Progression through each phase is controlled by cyclins (Ccn), cyclin-dependent kinases (Cdk) and Cyclin-dependent kinase inhibitors (CKIs) [67].

Our analysis showed a downregulation of the genes coding for Cyclin-dependent kinase inhibitors (CKIs) belonging to the INK4 protein family. In particular, *Cdkn2a*, *Cdkn2c* and *Cdkn2d* encode for p16^INK4a^, p18^INK4c^ and p19^INK4d^, respectively, which inhibit the activity of the Cdk/cyclin complex. Furthermore, p16^INK4a^, p18^INK4c^ and p19^INK4d^ regulate the quiescent state by their specific binding to Cdk4/6, thus preventing its interaction with Cyclin D [68]. The downregulation of p16^INK4a^ expression was experimentally confirmed by immunochemistry assay and densitometric analysis. On the basis of p16^INK4a^, p18^INK4c^ and p19^INK4d^ downregulation, we could hypothesize an activation of cell cycle-associated genes.

The immunochemistry assay and relative densitometric analysis highlighted Cyclin D1 expression in untreated differentiated NSC-34 cells and 20 µM CBN-treated differentiated NSC-34 cells. However, its expression level in 20 µM CBN-treated differentiated NSC-34 cells was lower compared to that in the untreated ones. Despite the downregulation of *Ccnd1*, *Ccnd3*, *Cdk4* and *Cdk6* in the early G1 phase, our data showed the upregulation of *Rb1*, *Rbl1*, *Ccne2* and *Cdk2*. *Rb1* and *Rbl1* encode for Rb and p107 proteins, and *Ccne2* and *Cdk2* encode for Cyclin E and Cdk2 proteins, all responsible for the G1 checkpoint. It has been demonstrated that Rb proteins are phosphorylated during progression through the G1 phase. In the late G1 phase, Cyclin E/Cdk2 complex phosphorylates Rb proteins, thus promoting the cell cycle progression towards the S phase [69,70].

Cyclin E has the important function of inducing the S phase and induces DNA replication by the expression of S phase specific genes. Furthermore, the non-canonical role of Cyclin E in terminally differentiated neurons has been demonstrated through proteomic analysis: Cyclin E influences synaptic plasticity by inhibiting Cdk5, and its downregulation in postmitotic neurons decreases the numbers of synapses and dendritic spines [28].

Proceeding through the cell cycle, the transcription of S phase-specific genes is regulated by the activation of E2F family proteins classified both as transcriptional activators and repressors. In particular, E2F1, E2F2 and E2F3 are well-established transcriptional activators [71], while E2F4, E2F6, E2F7 and E2F8 are categorized as transcriptional repressors [72]. E2F1, E2F2 and E2F3 are activated by dimerization with DP protein and drive the transition from G1 to S phase, enhancing cell proliferation [73]. In our study, genes encoding for E2f1 and E2f4 proteins were downregulated, whereas *E2f2*, *E2f3* and *Tfdp2* (coding for DP-2 protein) were upregulated.

Moreover, CBN was able to enhance the expression of the *Cdk7* gene, coding for a serine/threonine kinase classified as transcriptional factor. Furthermore, Cdk7 is a subunit of the transcriptional initiation factor II-H (TFIIH); thus, it plays a major role in transcription of immediate-early genes (IEGs). Previous studies ultimately demonstrated that Cdk7 is differentially expressed in adult brain and regulates the expression of many proteins involved in synaptic plasticity [30].

During the S phase of the cell cycle, the proteins encoded by *Dpf4* and *Cdc7* are required for activation of the MCM helicase and for the initiation of DNA replication at multiple origins throughout the genome [74]. These two genes were upregulated in our comparative analysis.

The phosphorylation of Cyclin B (encoded by *Ccnb1* gene) by Cdc25c (encoded by *Cdc25c* gene) determines the G2/M phase transition.

During interphase, Cdc25c has poor phosphorylation activity because of its cytoplasmatic localization, promoted by its binding with 14-3-3 proteins (encoded by *Ywhae*, *Ywhag*, *Ywhah* and *Ywhaq* genes) [59]. In our data, *Cdc25c* was upregulated, and the genes *Ywhae*, *Ywhag*, *Ywhah*, *Ywhaq* were downregulated. This suggests that Cdc25c is active; thus, this protein is likely localized in the nucleus. Additionally, Cdc25c activation requires its translocation into the nucleus and its dissociation from 14-3-3 proteins. Cdc25c is essential for regulating the cell cycle, and its activity can be inhibited by p53 and checkpoint protein kinases CHK1 and CHK2 in response to DNA damage [75]. Cdc25c inhibition prevents the activation of the cyclin B1/CDK1 complex, thus arresting the cell cycle in the G2/M phase [76]. Moreover, downregulation of Cdc25c was reported in the literature to ultimately lead to cell death [77].

During cell cycle progression, the precise oscillatory expression of cyclins and their inhibitors is finely regulated by the proteasomal mechanism involved in protein degradation. The anaphase-promoting complex/cyclosome (APC/C) is the main regulator of protein degradation during the cell cycle. The treatment with 20 µM of CBN triggered an upregulation of genes coding for proteins belonging to the APC/C complex (*Anapc11*, *Anapc10*, *Cdc23*, *Cdc16*, *Anapc4*, and *Cdc27*). The role of the APC/C complex is exerted through the temporal and spatial regulation of APC/C activity and its substrate specificity. In metaphase, the APC/C complex degrades two repressors of the anaphase transition, Securin and Cyclin B [78]. Securin is an inhibitor of Separase (coded by *Espl1* gene), a protease that cleaves the cohesion complex (encoded by *Stag1*, *Smc3*, *Smc1a*, *Nipbl*, *Pds5a*, *Pds5b*, and *Wapl* genes). The disassembly of the cohesion complex triggers sister chromatid segregation and the onset of anaphase [79]. Moreover, it has been observed that reduced levels of Cyclin B are required to enter anaphase [80]. Finally, the APC/C complex is highly expressed in postmitotic neurons and has been related to axon guidance, synaptic plasticity, neurogenesis, and neuronal survival [32,81].

In eukaryotic cells, cell cycle checkpoints are essential mechanisms that regulate DNA replication and repair, mitosis, and cytokinesis. They are crucial during the cell cycle for both the occurrence and conclusion of mitosis. Nonetheless, they ensure that critical events of a cell cycle phase are completed before entering the next one, thus coordinating cell growth with cell proliferation. If cells are arrested at any of the checkpoints, they will either return to G0 and re-differentiate or die by apoptosis [82]. It has been shown that at the checkpoint G1/S, E2f1 can regulate neuronal apoptosis. The suggested mechanisms underpinning this process include the activation of Bax/caspase in a p53-independent manner [83] and the activation of the Cdk1/FOXO1/Bad pathway [84]. The most important genes involved in cell death, such as *Bax*, *Caspase-3*, *Caspase-9*, *Caspase-8*, *Caspase-10* and *Bak*, showed no differential expression in our comparative transcriptomic analysis, meaning there were no substantial differences between 20 μM CBN and CTRL conditions. The pro-apoptotic gene *Bad* was instead downregulated by the treatment. As confirmed by the MTT assay, CBN regulates cell cycle-associated genes without simultaneously affecting the expression of genes associated with the apoptosis pathway.

To rule out the activation of other cell death processes, we investigated the dysregulation of the genes involved in the necroptosis and ferroptosis pathways.

Necroptosis is a type of programmed cell death that generally manifests with morphological features of necrosis. This type of cell death occurs after the activation of tumor necrosis receptor (Tnrf1) and Fas cell surface death receptor (Fas), triggered by Tnfα and FasL respectively; or through activation of Toll-like receptor 4 (Tlr4) and mixed lineage kinase domain-like (MLKL). These receptors activate a downstream serine/threonine protein kinase 1 (Ripk1) that induces the destruction of the cell membrane integrity, the swelling of the organelles and, consequently, cell death [85]. In our study, CBN did not activate the genes involved in this programmed cell death; specifically, necroptosis-associated genes *Tnfr1*, *Tnfα*, *Fas*, *FasL*, *Tlr4*, *Ripk1* and *Mlkl* were not differentially expressed after CBN exposure.

Finally, the ferroptosis pathway was also investigated. Ferroptosis is an iron-dependent cell death process caused by a redox imbalance between the production of oxidants and antioxidants, which results in the accumulation of lipid peroxidation and dysfunction of the antioxidant system. The main recognized mechanism involved in ferroptosis is related to a decrease in the activity of glutathione peroxidase 4 (Gpx4) [86] which is encoded by the *Gpx-4* gene; this gene was not differentially expressed in our computational analysis.

Furthermore, recent studies have shown that aberrant reactivation of the cell cycle could be involved in metastatic retinoblastoma [87]; thus, we expanded our study on genes involved in cancer processes. As reported in our transcriptomic results, tumor suppressor genes were more upregulated than oncogenes, while the majority of the latter were mainly downregulated.

Overall, CBN at a concentration of 20 µM was able to regulate genes associated with the cell cycle in differentiated NSC-34 cells without inducing cell death or tumorigenesis. As discussed above, the regulation of these genes is not related exclusively to the progression of the cell cycle, but may have a role in the mechanisms underlying synaptic plasticity in postmitotic neurons.

The strength of this work relies on the transcriptomic analysis methodology, which allowed the investigation of genes involved in many different pathways. Compared to other methods, transcriptomic analysis offers the advantage of enabling the simultaneous examination of several genes at once, while allowing a more exhaustive and comprehensive assessment of complex biological processes.

The main limitation of this study is represented by the lack of an in vitro disease model that could be implemented in future studies to confirm the suitability of CBN for the reactivation of cell cycle-associated genes in the context of neurodegeneration. Therefore, it would be interesting to perform additional experiments aimed at investigating the neuroprotective effect of CBN on NSC-34 cells after neurotoxic treatment or deprivation of neurotrophic factors.

## 5. Conclusions

In this preliminary study, we highlighted that CBN at a concentration of 20 µM regulates cell cycle-associated genes but not genes associated with cell death and cancer. In particular, the inhibitors of the cell cycle, *Cdkn2a*, *Cdkn2c* and *Cdkn2d* genes, were downregulated, while promoters of the cell cycle, *Ccne2*, *Cdk2*, *Cdk7*, *Anapc11*, *Anapc10*, *Cdc23*, *Cdc16*, *Anapc4*, *Cdc27*, *Stag1*, *Smc3*, *Smc1a*, *Nipbl*, *Pds5a*, *Pds5b*, and *Wapl* genes, were upregulated. It is interesting to note that these genes were found differentially expressed in the adult brain. Excluding *Cdk2*, these genes are required for the axonal maturation, migration and synaptic plasticity of neuron cells. These findings, supported by experimental evidence obtained from immunochemistry assays and relative densitometric analyses of p16^INK4a^ and Cyclin D1, suggest a potential role for CBN in the regulation of cell cycle-associated genes. The results obtained could be a starting point for testing CBN on models of motor neuron diseases characterized by synaptic dysfunctions and aberrant reactivation of the cell cycle leading to cell death.

## Figures and Tables

**Table 1:** Summary of six different gene ontologies that resulted in the top 30 enriched biological processes from clusterProfiler analysis. For each BP, the relative number of differentially expressed genes (DEGs count), both upregulated (Up DEGs count) and downregulated (Down DEGs count), related to cell cycle pathway, is reported. The Net effect column reports the behavior of each process considering only the cumulative fold change of the DEGs as well as their function. To note, the final effect may also be further regulated by factors not investigated in our analyses.

Gene Ontology Terms	DEGs Count	Up DEGs Count	Down DEGs Count	Hypothesized Net Effect
Chromosome segregation	31	21	10	Activated
Mitotic cell cycle phase transition	38	18	20	Activated
Mitotic nuclear division	29	18	11	Activated
Nuclear chromosome segregation	31	21	10	Activated
Regulation of cell cycle phase transition	35	17	18	Activated
Regulation of chromosome organization	24	13	11	Activated

## Data Availability

The data presented in this study are openly available in the NCBI sequence Read Archive under BioProject accession number PRJNA1078152.

## Supplementary Materials

The following supporting information can be downloaded at https://www.mdpi.com/article/10.3390/biomedicines12061340/s1; Figure S1: ^1^H NMR of CBN in CDCl_3_ (400 MHz) spectra; Figure S2: Heatmap of the genes that differentially expressed under each CBN concentration; Table S1: Table containing all DEGs resulting from our comparative expression analysis; Table S2: Table containing all DEGs involved in the cell cycle, with the corresponding cell cycle phase and subphase; Table S3: GO. BP. Enriched resulting from the comparison of 20 μM CBN vs. CTRL; Table S4: List of genes used for the evaluation of cell death processes; Table S5: List of oncogenes and tumor suppressor genes resulting from mouse genome informatics; Table S6: Cell cycle-related enriched BP ontologies.
