# Supplementary figures and images for: Cannabinol Regulates the Expression of Cell Cycle-Associated Genes in Motor Neuron-like NSC-34: A Transcriptomic Analysis
Source: Biomedicines. 2024 Jun 17;12(6):1340. doi: 10.3390/biomedicines12061340 (PMC11201772; doi:10.3390/biomedicines12061340)

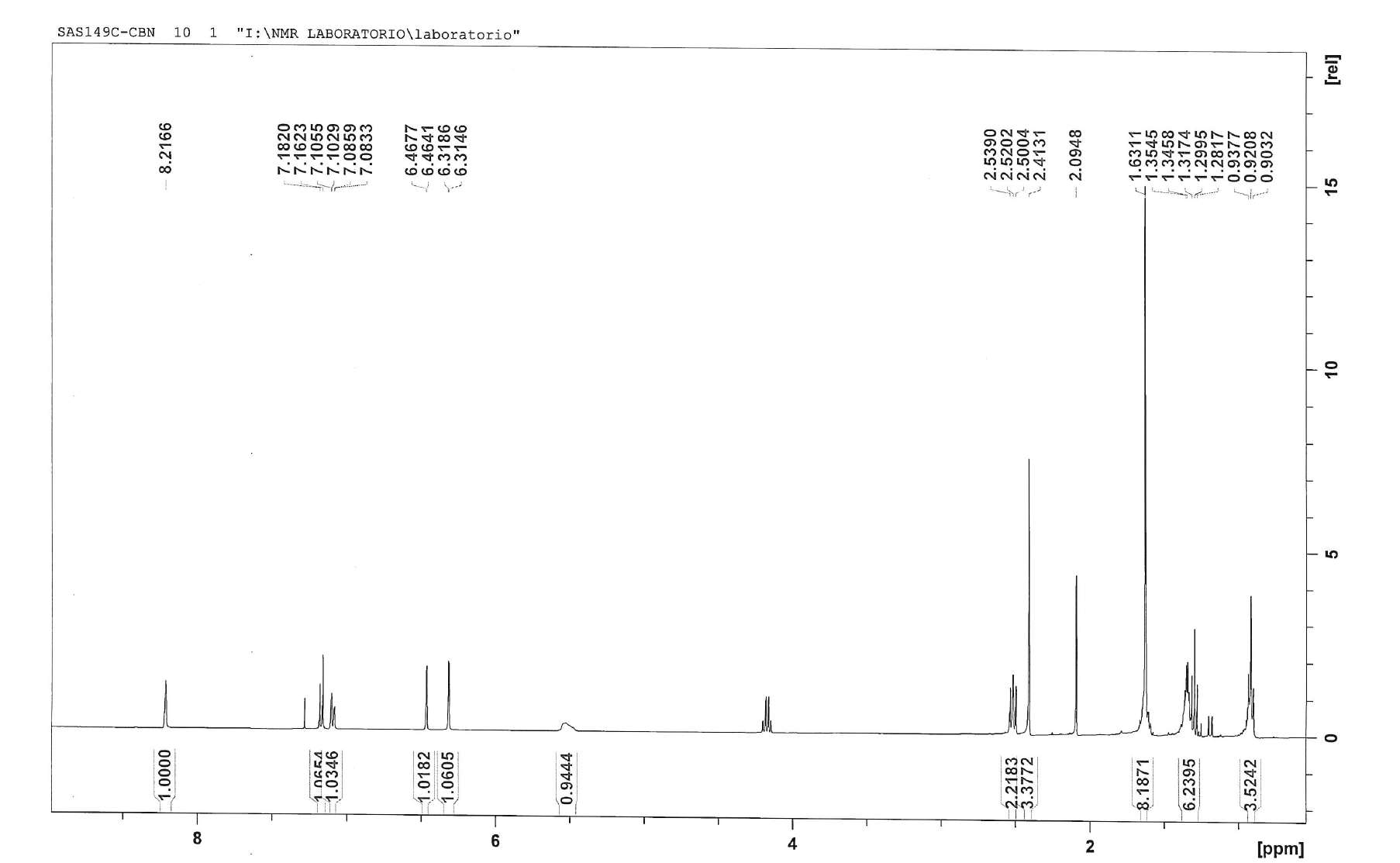

Supplement: Supplementary file 1 [file biomedicines-12-01340-s001.zip › Figure S1.tif]
